# Laparotomy and laparoscopy diversely affect macrophage-associated antimicrobial activity in a murine model

**DOI:** 10.1186/1471-2172-14-27

**Published:** 2013-06-20

**Authors:** Shun Gen Huang, Yi Ping Li, Qi Zhang, H Paul Redmond, Jiang Huai Wang, Jian Wang

**Affiliations:** 1 Department of Pediatric Surgery, Affiliated Children’s Hospital, Soochow University, Suzhou, China; 2 Department of Academic Surgery, University College Cork, Cork University Hospital, Cork, Ireland

**Keywords:** Laparotomy, Laparoscopy, Phagocytosis, Bactericidal activity, Innate immunity, Macrophages

## Abstract

**Background:**

Surgical intervention-related trauma contributes largely to the development of postoperative immunosuppression, with reduced resistance to secondary bacterial infection. This study compared the impact of laparotomy versus laparoscopy on macrophage-associated bactericidal ability and examined whether laparotomy renders the host more susceptible to microbial infection.

**Results:**

BALB/c mice were randomized into control, laparotomy, and laparoscopy groups. Laparotomy, but not laparoscopy, significantly downregulated CR3 expression on macrophages, diminished macrophage-induced uptake and phagocytosis of *E. coli* and *S. aureus*, and impaired macrophage-mediated intracellular bacterial killing. Consistent with this, mice that underwent laparotomy displayed substantially higher bacterial counts in the blood and visceral organs as well as a significantly enhanced mortality rate following bacterial infection, whereas mice subjected to laparoscopy did not show any defects in their bacterial clearance.

**Conclusion:**

Laparotomy has an adverse effect on host innate immunity against microbial infection by impairing macrophage-mediated phagocytosis and killing of the invaded bacteria. By contrast, laparoscopy appears to preserve macrophage-associated bactericidal ability, thus alleviating the development of postoperative immunosuppression.

## Background

The innate immune system plays a critical role in protecting the host against microbial infection, and this function relies on a rapid detection of pathogen-associated molecular patterns (PAMPs) present on microbial pathogens by Toll-like receptors (TLRs) and other pattern-recognition receptors (PRRs) expressed by innate immune cells such as monocytes/macrophages, with an ultimate inflammatory response [1,2]. Among the innate immunity-comprised host defense functions, phagocytosis of the invaded pathogens by professional phagocytes including macrophages and polymorphonuclear neutrophils (PMNs) is of crucial importance for the host to clear these pathogens from the body [3-5]. Phagocytosis is initiated by TLR-associated recognition of, followed by engulfment of the invading pathogens by phagocytes via phagocytic receptors, and once the phagosome is formed, the ingested microbial pathogens are degraded and killed within phagocytes through a process of phagosome/lysosome fusion [4-7].

Accumulated data have well-documented that surgical intervention-related trauma contributes to the development of postoperative immunosuppression that is responsible for an increased risk of secondary bacterial infection [8-13]. Surgical trauma primes and activates monocytes/macrophages and PMNs, and results in inflammatory mediator release from these cells. Although these mediators have a theoretically beneficial role, they may, if excessively released, lead to the systemic inflammatory response syndrome (SIRS), a condition that subsequently causes inhibition of host immune reactions, damage to cells and tissues, and end-organ dysfunction [14-17]. Watson et al. [18] found that open laparotomy significantly enhanced the release of the proinflammatory cytokine TNF-α and reactive oxygen-derived species superoxide anion from peritoneal macrophages, but markedly impaired macrophage-mediated phagocytic activity. In contrast, laparoscopy does not trigger an apparent systemic inflammatory response, largely as a result of reduced wound trauma [13,18-20]. Indeed, a clinical study has demonstrated better preservation of monocyte functions in patients undergoing laparoscopic versus open cholecystectomy [21].

Although open laparotomy has been shown to induce an unfavourable host immune response with increased inflammatory mediator release and decreased phagocytic function in macrophages, it is unclear whether open laparotomy has an adverse effect on macrophage-associated bactericidal activity. We hypothesized that open laparotomy leads to an impaired bactericidal ability in macrophages, thus rendering the host more susceptible to microbial infection. We report here that laparotomy in contrast to laparoscopy is associated with reduced phagocytic receptor expression, impaired macrophage-mediated bacterial phagocytosis and killing, and delayed bacterial clearance in a murine model, which may facilitate the development of microbial infection postoperatively via the established immunosuppressive state.

## Methods

### Mice and surgical procedures

Pathogen-free, 8- to 10-week old BALB/c mice were housed in barrier cages under controlled environmental conditions (12/12 h of light/dark cycle, 55% ± 5% humidity, 23°C) in the experimental animal center of Affiliated Children’s Hospital, Soochow University (Suzhou, China), and had free access to standard laboratory chow and water. All animal procedures were approved by the Animal Care and Use Committee of Soochow University before the experiment, and conducted under the Guidelines for Animal Care and Use of Soochow University.

Mice were fasted for 12 h before experiments but allowed water *ad libitum*, and randomized into one of the three groups: control, laparotomy, and laparoscopy. Mice in the control group underwent general anesthesia with intramuscular injection of 100 μl ketamine/xylazine admixture (150 μl ketamine + 150 μl xylazine made up to 1 ml with 0.9% saline) but no surgical procedures. Mice in the laparotomy group underwent a 2-cm midline abdominal incision under a sterile condition. The wound edges were retracted to allow for appropriate air exposure of the peritoneal contents for 15 min but without taking the gut out of the peritoneal cavity. The abdomen was then closed with 6/0 prolene sutures. Mice in the laparoscopy group had inflation of the peritoneal cavity with carbon dioxide using an 18-G needle through the lower midline of the abdomen, with a maximum pressure of 5 mmHg for 15 min, to create a sterile CO_2_ pneumoperitoneum.

### Detection of phagocytic receptor expression on peritoneal macrophages

Peritoneal lavage was collected from the control mice and mice that underwent laparotomy or laparoscopy 24 h after the surgical procedures, and dual stained with anti-F4/80 antigen (Serotec, Oxford, UK), anti-complement receptor type 3 (CR3) (BD PharMingen, San Diego, CA), and anti-FcγIII/II receptor (FcγR) (BD PharMingen) mAbs conjugated with PE or FITC. PE- or FITC-conjugated anti-mouse isotype-matched mAbs (Serotec) (BD PharMingen) were used as negative controls. Erythrocytes were lysed using lysis buffer (BD Biosciences). FACScan analysis was performed from at least 10,000 events to detect the surface expression of CR3 and FcγR on macrophages (F4/80-positive cells) using CellQuest software (BD Biosciences).

### Assessment of bacterial uptake and phagocytosis by peritoneal macrophages

FITC-conjugated gram-negative *Escherichia coli* (*E. coli*) and gram-positive *Staphylococcus aureus* (*S. aureus*) were obtained from Molecular Probes (Eugene, OR). Bacterial uptake that includes both the ingested bacteria and the bound but non-ingested bacteria, and bacterial phagocytosis that includes the ingested bacteria only were determined as described previously [22,23]. Briefly, peritoneal lavage was collected from the control mice and mice that underwent either laparotomy or laparoscopy 24 h after the surgical procedures and incubated with 1 × 10^6^ colony formation units (CFU)/ml of FITC-labeled *E. coli* or *S. aureus* at 37°C for 15 and 30 min. Bacterial uptake by peritoneal macrophages was assessed by FACScan analysis. Peritoneal macrophages were identified by their positive staining for anti-F4/80 antigen. Bacterial phagocytosis was further determined after the external fluorescence of the bound, but non-ingested, bacteria was quenched with 0.025% crystal violet (Sigma-Aldrich, St. Louis, MO).

### Determination of macrophage-mediated intracellular bacterial killing

Peritoneal lavage was collected as mentioned above. Macrophages in the peritoneal lavage were isolated by removal of the suspension cells after a 90-min adherence step. Purity of the isolated peritoneal macrophages was usually >95% as confirmed by FACScan analysis of the F4/80-positive cells.

Isolated peritoneal macrophages were chased with live *E. coli* or *S. aureus* (ATCC, Manassas, VA) at 37°C with a macrophage/bacteria ratio of 1:20 for 10 min, and this time point was marked as time 0. Thereafter, macrophages and bacteria were co-cultured at 37°C for further 30 and 60 min, and these time points were marked as time 30 and 60, respectively. After removal of the culture supernatants at each time point, macrophages were washed with PBS and lysed in 0.5 ml lysis buffer containing 0.5% Triton X-100 (Sigma-Aldrich) for 10 min. Serial 10-fold dilutions of the cell lysates were plated on lysogeny broth agar (Sigma-Aldrich) or trypticase soy agar (Merck, Darmstadt, Germany), and cultured for 24 h at 37°C for determination of bacterial CFU. Intracellular bacterial killing by macrophages, as represented by the bactericidal rate, was calculated using the following formula: bactericidal rate (%) = 1 – (CFU at time 30 or time 60 / CFU at time 0 × 100%).

Peritoneal macrophages isolated from the control mice and mice that underwent either laparotomy or laparoscopy 24 h after the surgical procedures were pretreated with 50 μM 1400 W (N-(3-(Aminomethyl)benzyl)acetamidine) (Sigma-Aldrich), a highly selective inducible nitric oxide synthase (iNOS) inhibitor for 4 h, and then chased with live *E. coli* or *S. aureus* at 37°C with a macrophage/bacteria ratio of 1:20 for 10 min. The bactericidal rate was assessed as described previously.

### Detection of intracellular nitric oxide formation in peritoneal macrophages

Peritoneal lavage was collected from the control mice and mice that underwent laparotomy or laparoscopy 24 h after the surgical procedures as mentioned above. Intracellular nitric oxide (NO) formation in peritoneal macrophages was detected by using the fluorescent probe 4-amino-5-methylamino-2′,7′-difluorescein (DAF-FM) diacetate (Molecular Probes) as described previously [23]. Briefly, peritoneal cells in the lavage were incubated with culture medium or 1 × 10^6^ CFU/ml heat-killed bacteria of either *E. coli* or *S. aureus* at 37°C for 30 min, and further loaded with 5 μM DAF-FM diacetate and stained with PE-conjugated anti-F4/80 antigen mAb (Serotec) for additional 30 min. Intracellular NO formation in peritoneal macrophages (F4/80-positive cells) was detected by FACScan analysis.

### Examination of bacterial clearance and animal survival in bacteria-infected mice

Gram-negative *E. coli* and gram-positive *S. aureus* were cultured at 37°C in LB broth (Sigma-Aldrich) or trypticase soy broth (Merck), harvested at the mid-logarithmic growth phase, washed twice, and resuspended in PBS for *in vivo* use. The concentration of resuspended bacteria was determined and adjusted spectrophotometrically at 550 nm.

To enumerate bacterial counts in the blood and visceral organs, mice in the control group and mice that underwent laparotomy or laparoscopy 24 h after the surgical procedures received an intraperitoneal injection of 200 μl PBS containing either live *E. coli* (0.75 × 10^7^ CFU/mouse) or live *S. aureus* (1 × 10^7^ CFU/mouse). All mice were killed at 24 and 48 h after bacterial infection by cervical dislocation. Blood samples were obtained by retinal artery puncture, and the dissected liver, spleen and lung were homogenized in sterile PBS. Serial 10-fold dilutions of whole blood and organ homogenates in sterile water containing 0.5% Triton X-100 were plated on LB agar or trypticase soy agar, and cultured for 24 h at 37°C for determination of bacterial CFU.

To compare the mortality rate after bacterial infection between different experimental groups, mice in the control group and mice that underwent either laparotomy or laparoscopy 24 h after the surgical procedures received an intraperitoneal injection of live *E. coli* (1.5 × 10^7^ CFU/mouse) or live *S. aureus* (2 × 10^7^ CFU/mouse). Survival was monitored for at least 14 days.

### Statistical analysis

All data are presented as mean ± SD. Statistical analysis was performed with GraphPad software, version 5.01 (Prism, La Jolla, CA). Comparison between groups was carried out using analysis of variance (ANOVA) for normally distributed data and Mann–Whitney *U* test for non-parametric data. Differences were judged statistically significant when the *p* value was less than 0.05.

## Results

### The effect of surgery on phagocytic receptor expression

**Figure 1:**
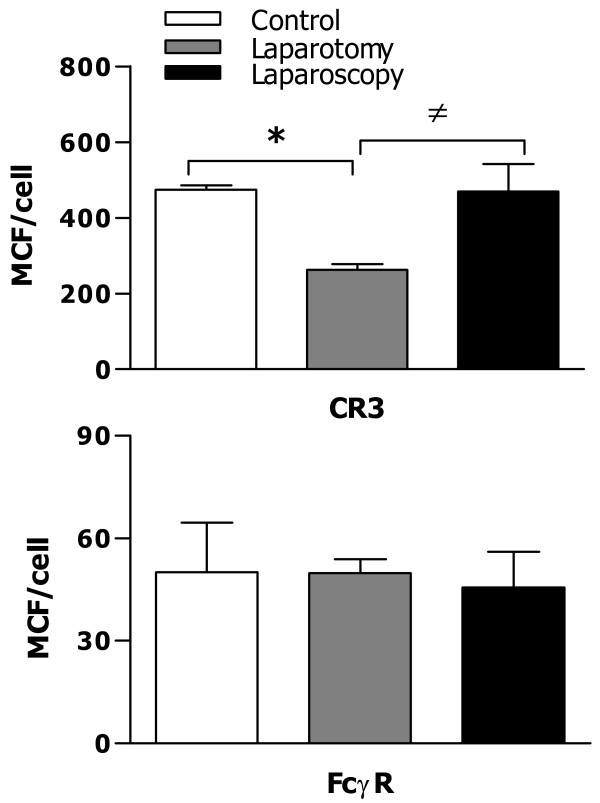
**Expression of phagocytic receptors CR3 and FcγR on peritoneal macrophages among control, laparotomy, and laparoscopy groups.** Peritoneal lavage was collected from the control mice (n = 8) and mice that underwent laparotomy (n = 8) or laparoscopy (n = 8) 24 h after the surgical procedures. Surface expression of CR3 and FcγR on peritoneal macrophages was assessed by FACScan analysis and expressed as mean channel fluorescence (MCF) per cell. Data are presented as mean ± SD. *p < 0.05 compared with control mice, ^≠^*p* < 0.05 compared with mice that underwent laparotomy.

To assess the impact of laparotomy versus laparoscopy on phagocyte-associated antimicrobial activity, we first measured the surface expression of two phagocytic receptors, CR3 and FcγR, on peritoneal macrophages collected from mice subjected to either laparotomy or laparoscopy 24 h after the surgical procedures. FACScan analysis demonstrated that laparotomy led to significantly downregulated expression of CR3 on macrophages when compared with the control mice (*p* = 0.0104) (Figure 1), whereas macrophages from mice that underwent laparoscopy did not show any reduction in their CR3 expression, with similar levels to those observed in macrophages from the control mice (Figure 1). In contrast to the CR3 expression, there were no significant differences in FcγR expression on macrophages found in mice among control, laparotomy, and laparoscopy groups (Figure 1).

### The effect of surgery on macrophage-mediated bacterial uptake, ingestion, and intracellular killing

**Figure 2:**
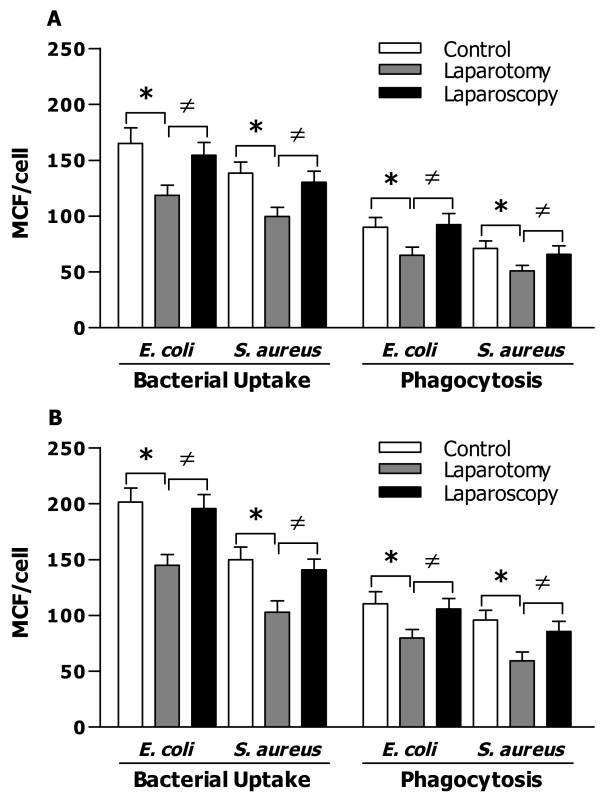
**Bacterial uptake and phagocytosis by peritoneal macrophages in the control mice and mice that underwent laparotomy or laparoscopy.** Bacterial uptake and phagocytosis were assessed after peritoneal macrophages were incubated with heat-killed *E. coli* or *S. aureus* for 15 (**A**) and 30 (**B**) min and compared between control (n = 14), laparotomy (n = 14), and laparoscopy (n = 14) groups. Data are mean ± SD of triplicate samples from at least five to seven independent experiments and expressed as mean channel fluorescence (MCF) per cell. *p < 0.05 compared with control mice, ^≠^*p* < 0.05 compared with mice that underwent laparotomy.

Next we examined whether surgery (laparotomy versus laparoscopy) influences macrophage-mediated uptake, phagocytosis, and intracellular killing of gram-negative *E. coli* and gram-positive *S. aureus*. Uptake and phagocytosis of either *E. coli* or *S. aureus* by macrophages from mice that underwent laparotomy were significantly less than those observed in macrophages from the control mice at 15 min post macrophage and bacteria co-cultures (*p* < 0.05), whereas macrophages from mice that underwent laparoscopy had similar levels of bacterial uptake and ingestion to those seen in macrophages from the control mice (Figure 2A). At 30 min post macrophage and bacteria co-cultures, significantly reduced uptake and phagocytosis of *E. coli* or *S. aureus* by macrophages were also evident in the laparotomy group (*p* < 0.05 versus both control and laparoscopy groups) (Figure 2B).

**Figure 3:**
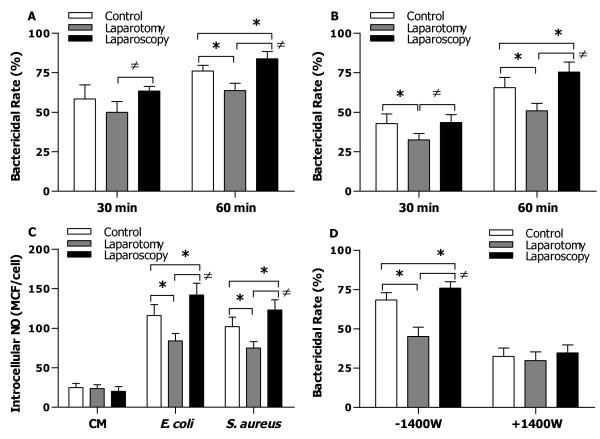
**Comparison of macrophage-mediated bactericidal activity and intracellular NO production between control, laparotomy, and laparoscopy groups.** Peritoneal macrophages were isolated from control mice (n = 28) and mice that underwent laparotomy (n = 28) or laparoscopy (n = 28). Intracellular bacterial killing by peritoneal macrophages, as represented by the bactericidal rate (%), was determined at 30 and 60 min after macrophages were chased with live *E. coli* (**A**) or *S. aureus* (**B**). Intracellular NO formation in peritoneal macrophages was detected by FACScan analysis after macrophages were incubated with culture medium (CM) and heat-killed *E. coli* or *S. aureus* for 30 min and expressed as mean channel fluorescence (MCF) per cell (**C**). Bactericidal rate was assessed at 60 min after peritoneal macrophages pretreated with culture medium or the iNOS inhibitor 1400 W (50 μM) for 4 h were chased with live *E. coli* (**D**). Data are mean ± SD of triplicate samples from at least four to six independent experiments. *p < 0.05 compared with control mice, ^≠^*p* < 0.05 compared with mice that underwent laparotomy.

Macrophages collected from mice that underwent laparotomy also displayed substantially diminished intracellular killing of both the ingested live *E. coli* (Figure 3A) and *S. aureus* (Figure 3B), as represented by significantly reduced bactericidal rates at 30 and 60 min after being chased with bacteria (*p* < 0.05 versus macrophages from the control mice), indicating that laparotomy results in an impaired antimicrobial activity in phagocytes. By contrast, laparoscopy did not have an adverse effect on macrophage-mediated intracellular bacterial killing; rather it strongly enhanced bactericidal rate at 60 min after the co-culture of macrophages with live *E. coli* or *S. aureus* when compared with macrophages from the control mice (*p* = 0.0417; *p* = 0.0442) and mice that underwent laparotomy (*p* = 0.0274; *p* = 0.0225) (Figure 3A and 3B).

There were no significant differences found in basal intracellular NO levels of macrophages among control, laparotomy, and laparoscopy groups (Figure 3C). Intracellular NO formation in response to gram-negative *E. coli* or gram-positive *S. aureus* was dramatically reduced in macrophages from mice that underwent laparotomy when compared with macrophages from the control mice (*p* = 0.0322; *p* = 0.397) (Figure 3C), whereas macrophages from mice that underwent laparoscopy showed significantly higher intracellular NO formation than that observed not only in macrophages from mice that underwent laparotomy (*p* = 0.0196; *p* = 0.0283) but also in macrophages from the control mice (*p* = 0.0402; *p* = 0.0436) (Figure 3C). Furthermore, blockage of iNOS activity with the highly selective iNOS inhibitor, 1400 W strongly attenuated macrophage-associated bactericidal capacity in response to gram-negative *E. coli* with no significant differences in bactericidal rate among control, laparotomy, and laparoscopy groups (Figure 3D). Similar results were also seen when 1400 W-pretreated macrophages were challenged with gram-positive *S. aureus* (data not shown). These data suggest that the impaired bactericidal activity of macrophages observed in mice that underwent laparotomy is, at least in part, due to the reduced intracellular NO formation.

### The effect of surgery on bacterial clearance and survival in a murine model of gram-negative or gram-positive infection

**Figure 4:**
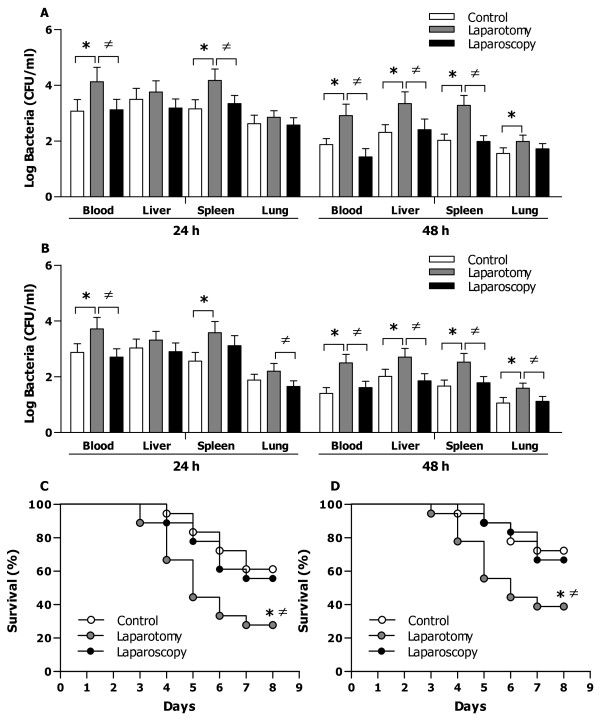
**Bacterial clearance and survival in bacteria-infected control mice and mice that underwent laparotomy or laparoscopy.** Control mice and mice that underwent laparotomy or laparoscopy were challenged with live *E. coli* (0.75 × 10^7^ CFU/mouse) (**A**) or *S. aureus* (1 × 10^7^ CFU/mouse) (**B**). Bacterial counts were determined in the blood, liver, spleen, and lung collected at 24 and 48 h post bacterial infection and expressed as Log CFU/ml. Data are mean ± SD of five to six mice per time point. *p < 0.05 compared with control mice, ^≠^*p* < 0.05 compared with mice that underwent laparotomy. Kaplan-Meier survival curves after control mice and mice that underwent laparotomy or laparoscopy challenged with live *E. coli* (1.5 × 10^7^ CFU/mouse, n = 18 per group) (**C**) or *S. aureus* (2 × 10^7^ CFU/mouse, n = 18 per group) (**D**). *p = 0.0176 (**C**) and *p = 0.0313 (**D**) compared with control mice, ^≠^*p* = 0.0395 (**C**) and ^≠^*p* = 0.0321 (**D**) compared with mice that underwent laparoscopy.

As macrophages collected from mice that underwent laparotomy were characterized with substantially reduced phagocytic receptor CR3 expression, diminished bacterial phagocytosis, and impaired bactericidal activity, we further determined whether laparotomy renders these mice more susceptible to bacterial infection. Bacterial CFU at 24 h post gram-negative *E. coli* (Figure 4A) or gram-positive *S. aureus* (Figure 4B) challenge was significantly greater in the blood (*p* < 0.05) and the spleen (*p* < 0.05) of mice subjected to laparotomy compared with the control mice. At 48 h post *E. coli* (Figure 4A) or *S. aureus* (Figure 4B) challenge, significantly higher bacterial counts were observed in the blood and all visceral organs measured including liver, spleen and lungs of mice subjected to laparotomy (*p* < 0.05 versus the control mice). These results indicate that laparotomy leads to a delayed clearance of the infected bacteria from these mice. Consistent with a preserved macrophage-associated bactericidal activity, mice that underwent laparoscopy had comparable levels of bacterial CFU in the circulation and visceral organs to those observed in the control mice (Figure 4A and 4B).

In response to gram-negative *E. coli* challenge at a lethal dose of 1.5 × 10^7^ CFU per mouse, control mice had an overall survival of 61%, whereas mice that underwent laparotomy showed a substantially reduced survival rate at 28% (*p* = 0.0176 versus control mice) (Figure 4C). Similarly, mice subjected to laparotomy were also more susceptible to gram-positive *S. aureus* infection with an overall survival of 39% compared with a 72% survival rate in the control mice (*p* = 0.0313) (Figure 4D). By contrast, the mortality rate in mice that underwent laparoscopy after live *E. coli* or *S. aureus* challenge was identical to that seen in the control mice (Figure 4C and 4D).

## Discussion

It has been shown that major open surgery results in an immunosuppressed state [8-13], which may lead to an increased risk of the host to develop secondary microbial infection postoperatively. In addition, suppressed host immune functions after surgery contribute positively to enhanced local and distant metastatic tumor growth in the setting of a neoplasm [24-26]. By contrast, the laparoscopic approach, since being introduced in the early 1990s, has been demonstrated to cause a significantly reduced systemic inflammatory response with better preserved host immune homeostasis than conventional laparotomy [10,13,18-21,27], which may account for the observed lower morbidity rates and faster recovery in patients after laparoscopic surgery compared to open surgery. Several factors and mechanisms have been postulated to be responsible for the occurrence of early postoperative immunosuppression after major open surgery [8-13,18-21,28,29], for example, the severity of surgical intervention-related trauma, an inappropriate systemic inflammatory response, depressed cell-mediated immune reactions, and the contamination and translocation of gram-negative bacterial lipopolysaccharide (LPS). Although the severity of wound trauma following open surgery is thought to be the primary cause for initiating an overwhelming inflammatory response and subsequently suppressed host immune reactions [8-13], open laparotomy-introduced LPS contamination in the peritoneal cavity and gut-derived LPS translocation have been shown to be responsible for postoperative immunological alterations [18]. A wide range of depressed cell-mediated immune functions including chemotaxis of PMNs, macrophage-associated immune responses, natural killer cell activity, lymphocyte proliferation and function has been observed following open surgery and is the feature of surgery-induced postoperative immunosuppression [9-13,18-21,28,29].

Monocytes/macrophages and PMNs are the professional phagocytes of the innate immune system and form the first line of host defense against the invading microbial pathogens [1-3]. Therefore, among surgery-suppressed cell-mediated immune functions, the influence of open laparotomy on phagocyte-associated antimicrobial activity plays a crucial role in determining the risk of developing bacterial infection postoperatively. In the present study, we first assessed phagocytic receptor CR3 and FcγR expression on peritoneal macrophages collected from the control mice and mice subjected to laparotomy or laparoscopy. Although laparotomy had no effect on FcγR expression, it caused significantly reduced expression of CR3 on macrophages. In contrast, laparoscopy affected neither FcγR nor CR3 expression. Both CR3 and FcγR contribute predominantly to phagocyte-associated uptake, ingestion, and killing of the invaded microbial pathogens [4,5]. Therefore, any defects in CR3 and/or FcγR may lead to a downregulated antimicrobial response, whereas overexpression of these receptors results in increased bacterial clearance [30]. As the macrophages collected from mice that underwent laparotomy had a reduced CR3 expression, we postulated that these macrophages may also suffer an impaired antimicrobial activity. Consistent with downregulated phagocytic receptor expression, macrophages collected from mice that underwent laparotomy displayed defects in bactericidal ability with not only significantly reduced bacterial uptake and phagocytosis but also markedly diminished intracellular killing of both the ingested *E. coli* and *S. aureus*.

Macrophage-produced NO contributes to the initial bactericidal activity against intracellular pathogens and peritoneal macrophages derived from iNOS knockout mice displayed impaired intracellular killing of *S. typhimurium*[31,32]. Indeed, blockage of iNOS with the selective iNOS inhibitor in the present study substantially attenuated macrophage-mediated bactericidal activity, suggesting that the reduced intracellular bacterial killing observed in macrophages collected from mice that underwent laparotomy may result, at least in part, from the diminished intracellular NO generation. Notably, laparoscopy, in addition to maintaining the levels of phagocytic receptor expression and bacterial ingestion, augmented macrophage-associated bacterial killing. Furthermore, in response to bacterial challenges macrophages from mice subjected to laparoscopy produced significantly more NO than macrophages from the control mice did, which may be responsible for the increased bactericidal rate observed in the laparoscopy group. However, the precise mechanisms by which laparoscopy causes increased intracellular NO production and subsequently enhanced bacterial killing in macrophages are still unclear.

Consistent with significantly reduced phagocytic receptor expression, diminished bacterial phagocytosis, and impaired bactericidal ability, mice subjected to laparotomy, in contrast to mice that underwent laparoscopy, displayed substantially delayed and reduced bacterial clearance from the circulation and visceral organs in response to either live *E. coli* or live *S. aureus* challenge, thus rendering these mice more susceptible to microbial infection. Notably, mice that underwent laparotomy showed a significantly enhanced mortality rate after gram-negative or gram-positive bacterial infection. Although the severity of laparotomy-associated surgical trauma, initiation of an inappropriate systemic inflammatory response, and contamination and translocation of LPS may all collectively offer a conceivable explanation [8-13,18], the precise mechanism underlying the apparently diverse effects induced by laparotomy versus laparoscopy on macrophage-associated antimicrobial activity has not yet been identified. Another limitation of the present study is that the impaired macrophage-mediated bactericidal activity observed in mice that underwent laparatomy has to be validated in the human setting as there are apparently different immune systems and responses between mice and humans.

## Conclusion

Our data indicate that laparotomy has an adverse effect on host innate immunity against microbial infection by reducing phagocytic receptor expression and impairing macrophage-associated bacterial ingestion and killing. On the other hand, laparoscopy not only maintains phagocytic receptor expression on macrophages but also preserves the bactericidal ability of macrophages, thus further supporting the evidence for recommendation of laparoscopic surgery over conventional laparotomy in reducing postoperative infection.

## Abbreviations

CFU: Colony formation units; CR3: Complement receptor type 3; FcγR: FcγIII/II receptor; iNOS: Inducible NO synthase; LPS: Lipopolysaccharide; MCF: Mean channel fluorescence; NO: Nitric oxide; PAMPs: Pathogen-associated molecular patterns; PMNs: Polymorphonuclear neutrophils; PRRs: Pattern-recognition receptors; SIRS: Systemic inflammatory response syndrome; TLRs: Toll-like receptors.

## Competing interest

The authors declare that they have no competing interests.

## Authors’ contributions

CGH, YPL, HPR, JHW, JW designed the study; CGH, YPL, QZ performed research; CGH, YPL, HPR, JHW analyzed data; CGH, JHW, JW wrote the paper. All authors read and approved the final manuscript.

## References

[B1] Akira S Uematsu S Takeuchi O Pathogen recognition and innate immunity Cell 2006 124 783 801 10.1016/j.cell.2006.02.015 16497588

[B2] Medzhitov R Recognition of microorganisms and activation of the immune response Nature 2007 449 819 826 10.1038/nature06246 17943118

[B3] Stuart LM Ezekowitz RAB Phagocytosis: elegant complexity Immunity 2005 22 539 550 10.1016/j.immuni.2005.05.002 15894272

[B4] Ehlers MR CR3: a general purpose adhesion-recognition receptor essential for innate immunity Microbes Infect 2000 2 289 294 10.1016/S1286-4579(00)00299-9 10758405

[B5] Vidarsson G van der Winkel JG Fc receptor and complement receptor-mediated phagocytosis in host defence Curr Opin Infect Dis 1998 11 271 278 10.1097/00001432-199806000-00002 17033391

[B6] Kinchen JM Ravichandran KS Phagosome maturation: going through the acid test Nat Rev Mol Cell Biol 2008 9 781 795 10.1038/nrm2515 18813294 PMC2908392

[B7] Blander JM Medzhitov R On regulation of phagosome maturation and antigen presentation Nat Immunol 2006 7 1029 1035 10.1038/ni1006-1029 16985500

[B8] Slade MS Simmons RL Yunis E Greenberg LJ Immunodepression after major surgery in normal patients Surgery 1975 78 363 372 1098195

[B9] Berti Riboli E Terrizzi A Arnulfo G Bertoglio S Immunosuppressive effect of surgery evaluated by the multitest cell-mediated immunity system Can J Surg 1984 27 60 63 6467104

[B10] Lee SW Feingold DL Carter JJ Peritoneal macrophage and blood monocyte functions after open and laparoscopic-assisted cecectomy in rats Surg Endosc 2003 17 1996 2002 10.1007/s00464-003-8154-5 14569448

[B11] Stephan RN Saizawa M Conrad PJ Depressed antigen presentation function and membrane interleukin-1 activity of peritoneal macrophages after laparotomy Surgery 1987 102 147 154 2956717

[B12] Lennard TW Shenton BK Borzotta A The influence of surgical operations on components of the human immune system Br J Surg 1985 72 771 776 10.1002/bjs.1800721002 2412626

[B13] Nguyen NT Luketich JD Schatz S Effect of open and laparoscopic surgery on cellular immunity in a swine model Surg Laparosc Endosc Percutan Tech 1999 9 176 180 10803994

[B14] Lenz A Franklin GA Cheadle WG Systemic inflammation after trauma Injury 2007 38 1336 1345 10.1016/j.injury.2007.10.003 18048040

[B15] Robertson CM Coopersmith CM The systemic inflammatory response syndrome Microbes Infect 2006 8 1382 1389 10.1016/j.micinf.2005.12.016 16679040

[B16] Gogos CA Drosou E Bassaris HP Skoutelis A Pro-versus anti-inflammatory cytokine profile in patients with severe sepsis: a marker for prognosis and future therapeutic options J Infect Dis 2000 181 176 180 10.1086/315214 10608764

[B17] van Deuren M van der Ven-Jongekrijg J Bartelink AK Correlation between proinflammatory cytokines and anti-inflammatory mediators and the severity of disease in meningococcal infections J Infect Dis 1995 172 433 439 10.1093/infdis/172.2.433 7622886

[B18] Watson RW Redmond HP McCarthy J Burke PE Bouchier-Hayes D Exposure of the peritoneal cavity to air regulates early inflammatory responses to surgery in a murine model Br J Surg 1995 82 1060 1065 10.1002/bjs.1800820820 7648154

[B19] Whelan RL Franklin M Holubar SD Postoperative cell mediated immune response is better preserved after laparoscopic vs open colorectal resection in humans Surg Endosc 2003 17 972 978 10.1007/s00464-001-8263-y 12640542

[B20] Allendorf JD Bessler M Whelan RL Better preservation of immune function after laparoscopic-assisted vs. open bowel resection in a murine model Dis Colon Rectum 1996 39 Suppl S67 S72 8831550 10.1007/BF02053809

[B21] Redmond HP Watson RW Houghton T Immune function in patients undergoing open vs laparoscopic cholecystectomy Arch Surg 1994 129 1240 1246 10.1001/archsurg.1994.01420360030003 7986152

[B22] Wang JH Doyle M Manning BJ Cutting edge: bacterial lipoprotein induces endotoxin-independent tolerance to septic shock J Immunol 2003 70 14 18 12496376 10.4049/jimmunol.170.1.14

[B23] O’Brien GC Wang JH Redmond HP Bacterial lipoprotein induces resistance to gram-negative sepsis in TLR4-deficient mice via enhanced bacterial clearance J Immunol 2005 74 1020 1026 15634926 10.4049/jimmunol.174.2.1020

[B24] Allendorf JD Bessler M Horvath KD Increased tumor establishment and growth after open vs laparoscopic surgery in mice may be related to differences in postoperative T-cell function Surg Endosc 1999 13 233 235 10.1007/s004649900952 10064753

[B25] Da Costa ML Redmond HP Finnegan N Flynn M Bouchier-Hayes D Laparotomy and laparoscopy differentially accelerate experimental flank tumour growth Br J Surg 1998 85 1439 1442 10.1046/j.1365-2168.1998.00853.x 9782033

[B26] Carter JJ Feingold DL Kirman I Laparoscopic-assisted cecectomy is associated with decreased formation of postoperative pulmonary metastases compared with open cecectomy in a murine model Surgery 2003 134 432 436 10.1067/S0039-6060(03)00136-3 14555930

[B27] Balagué C Targarona EM Pujol M Peritoneal response to a septic challenge. Comparison between open laparotomy, pneumoperitoneum laparoscopy, and wall lift laparoscopy Surg Endosc 1999 13 792 796 10.1007/s004649901101 10430687

[B28] Christou NV Superina R Broadhead M Meakins JL Postoperative depression of host resistance: determinants and effect of peripheral protein-sparing therapy Surgery 1982 92 786 792 7123498

[B29] Iwanaka T Arkovitz MS Arya G Ziegler MM Evaluation of operative stress and peritoneal macrophage function in minimally invasive operations J Am Coll Surg 1997 184 357 363 9100680

[B30] Weighardt H Feterowski C Veit M Increased resistance against acute polymicrobial sepsis in mice challenged with immunostimulatory CpG oligodeoxynucleotides is related to an enhanced innate effector cell response J Immunol 2000 165 4537 4543 11035094 10.4049/jimmunol.165.8.4537

[B31] Vazquez-Torres A Jones-Carson J Mastroeni P Ischiropoulos H Fang FC Antimicrobial actions of the NADPH phagocyte oxidase and inducible nitric oxide synthase in experimental salmonellosis. I. Effects on microbial killing by activated peritoneal macrophages in vitro J Exp Med 2000 192 227 236 10.1084/jem.192.2.227 10899909 PMC2193262

[B32] Mastroeni P Vazquez-Torres A Fang FC Antimicrobial actions of the NADPH phagocyte oxidase and inducible nitric oxide synthase in experimental salmonellosis. II. Effects on microbial proliferation and host survival in vivo J Exp Med 2000 192 237 248 10.1084/jem.192.2.237 10899910 PMC2193252

